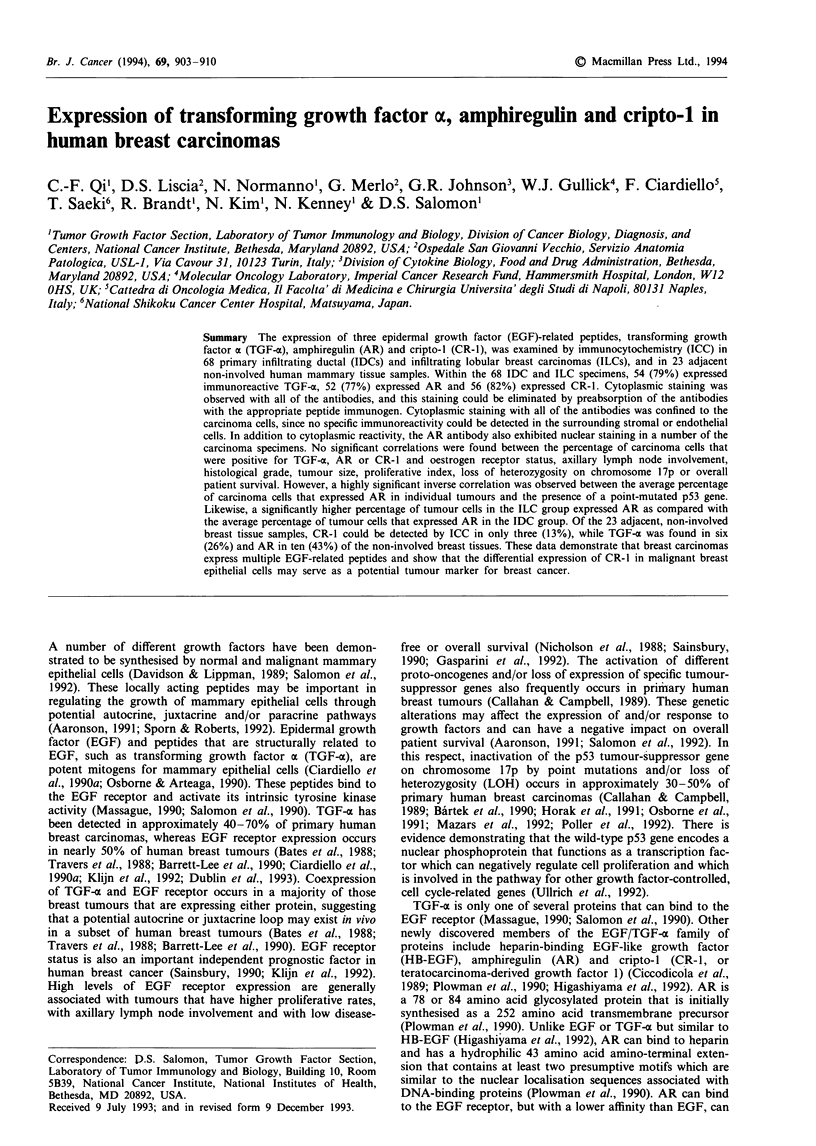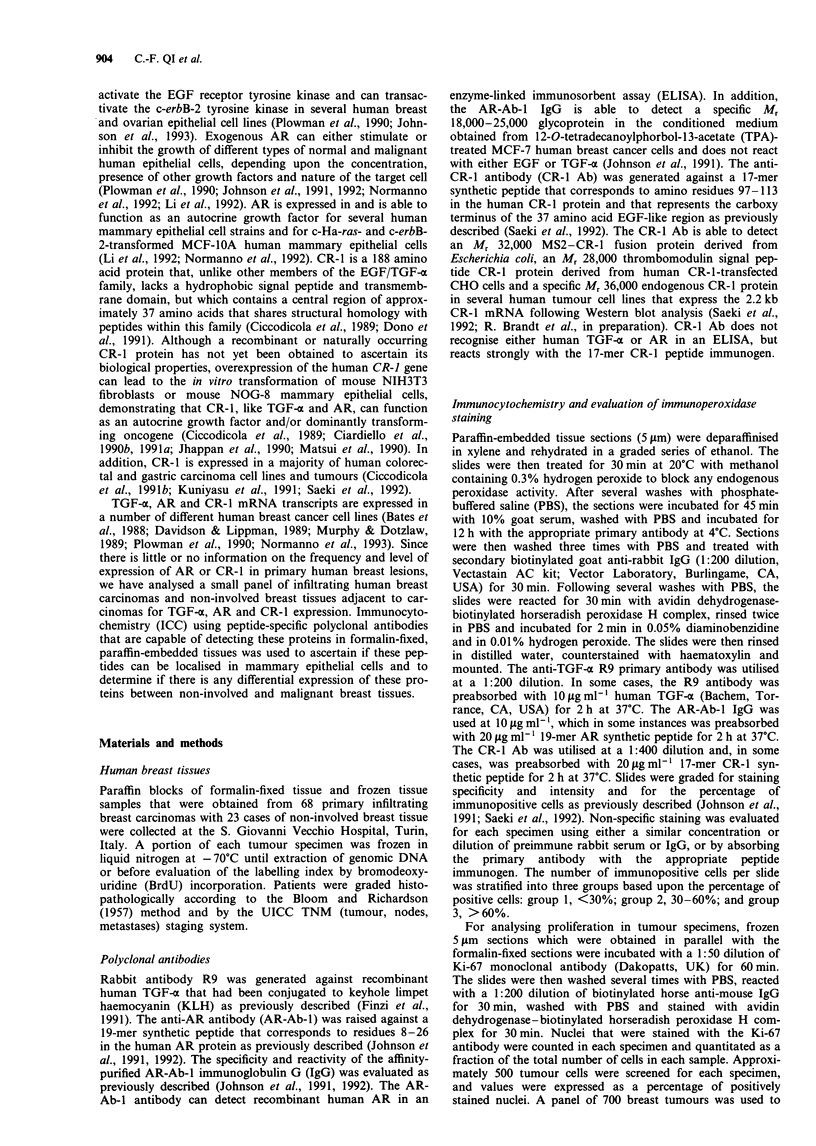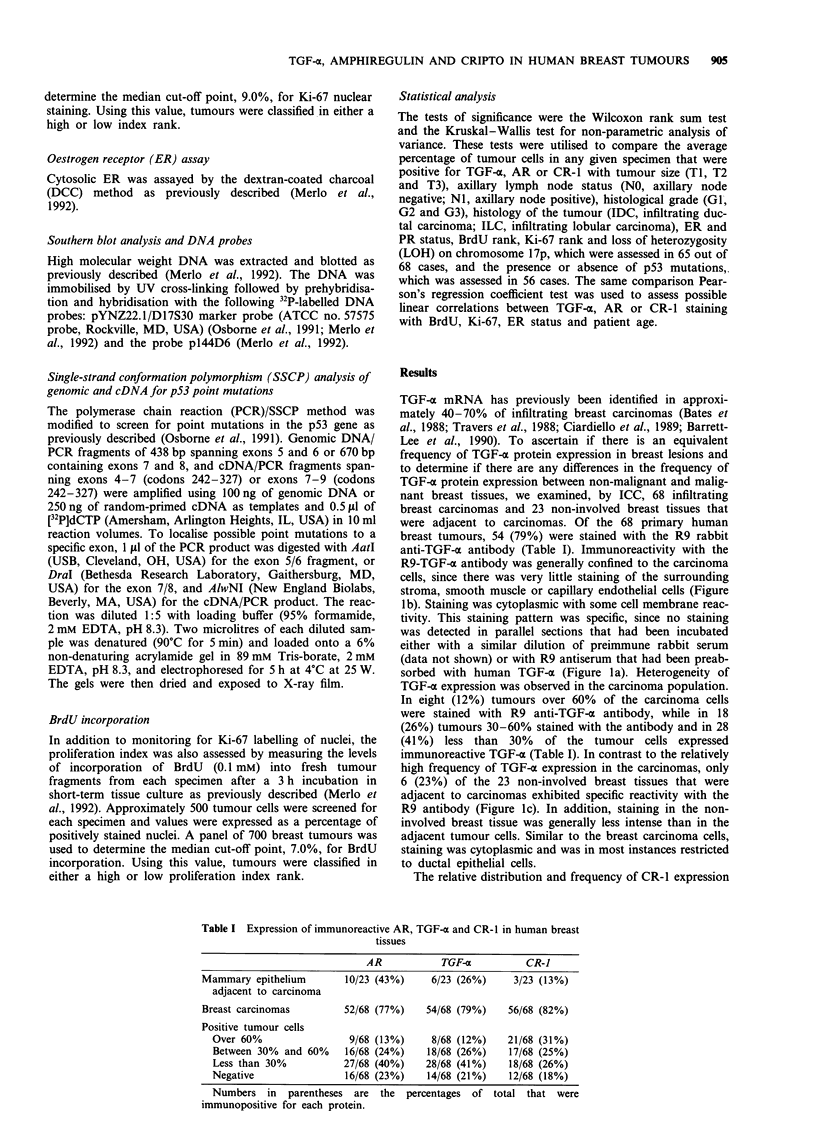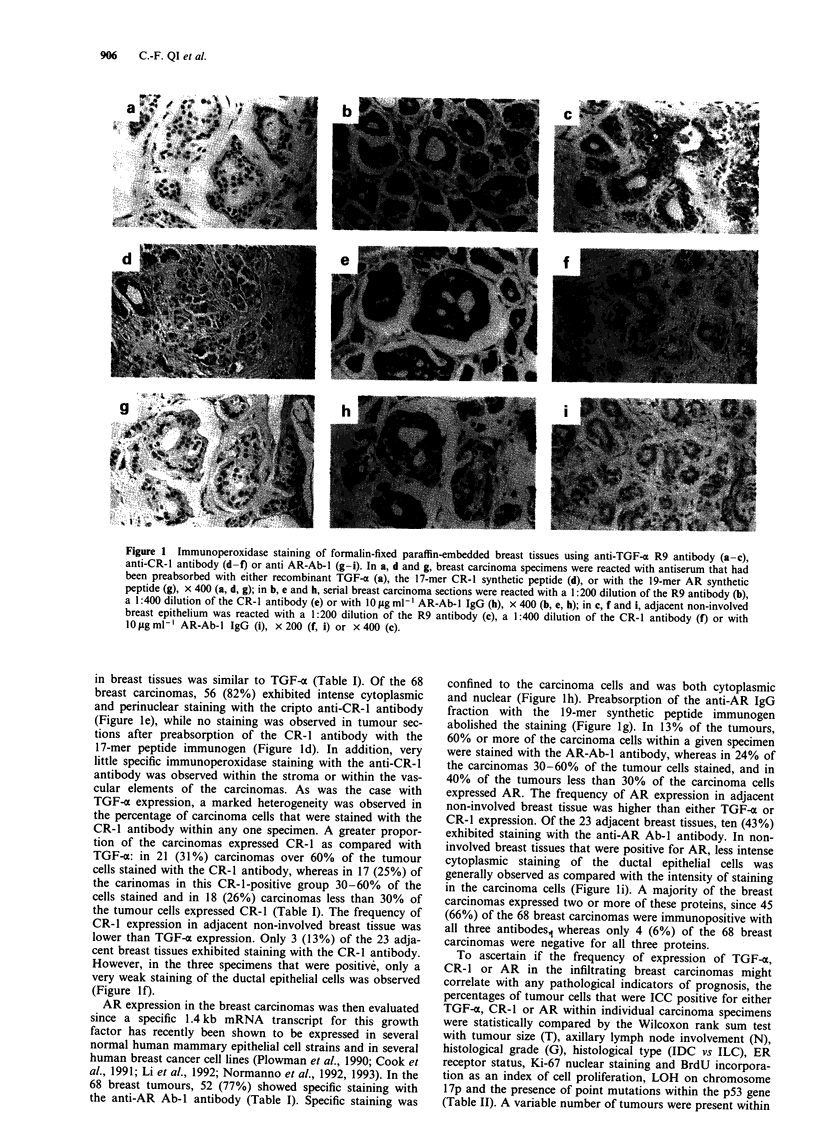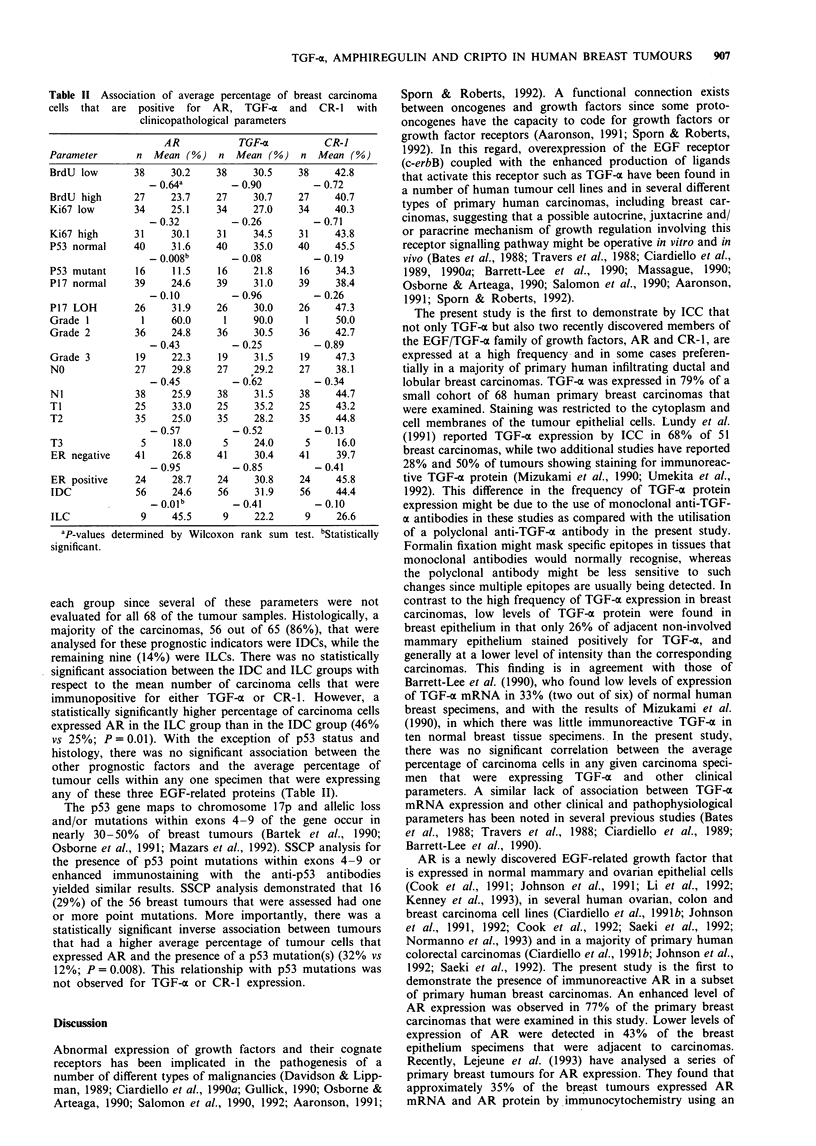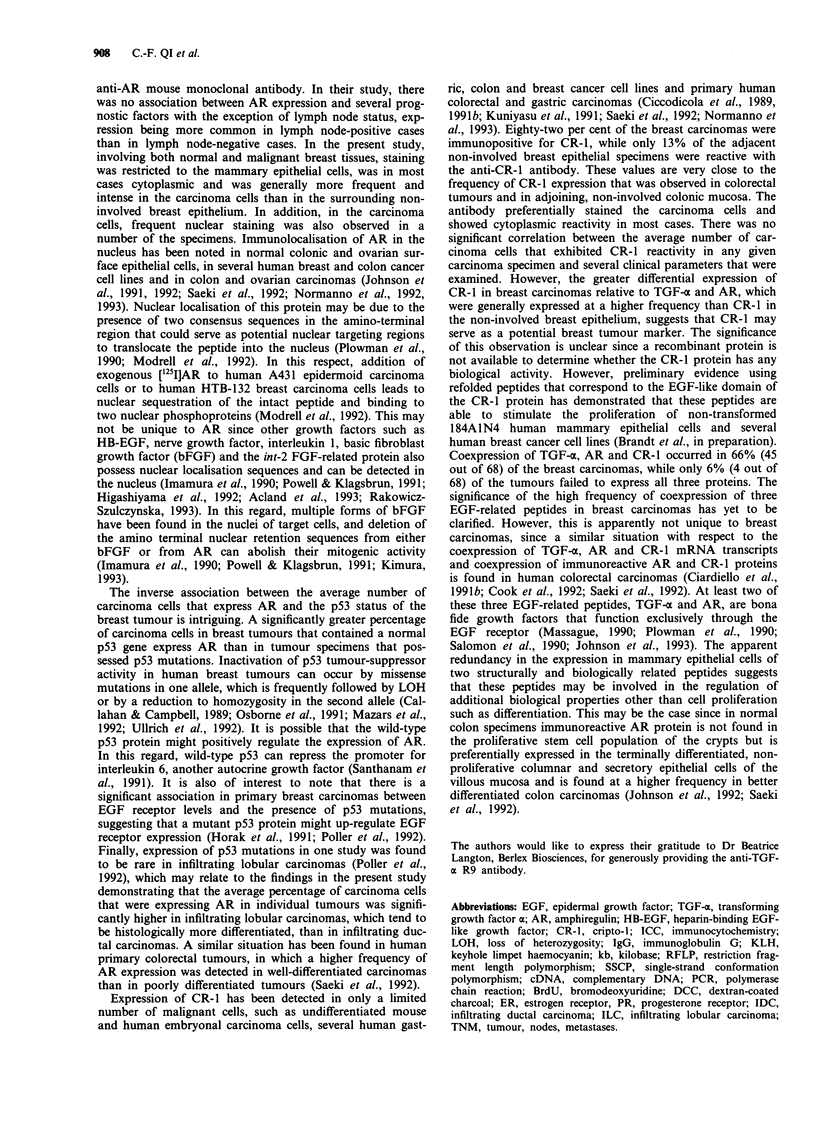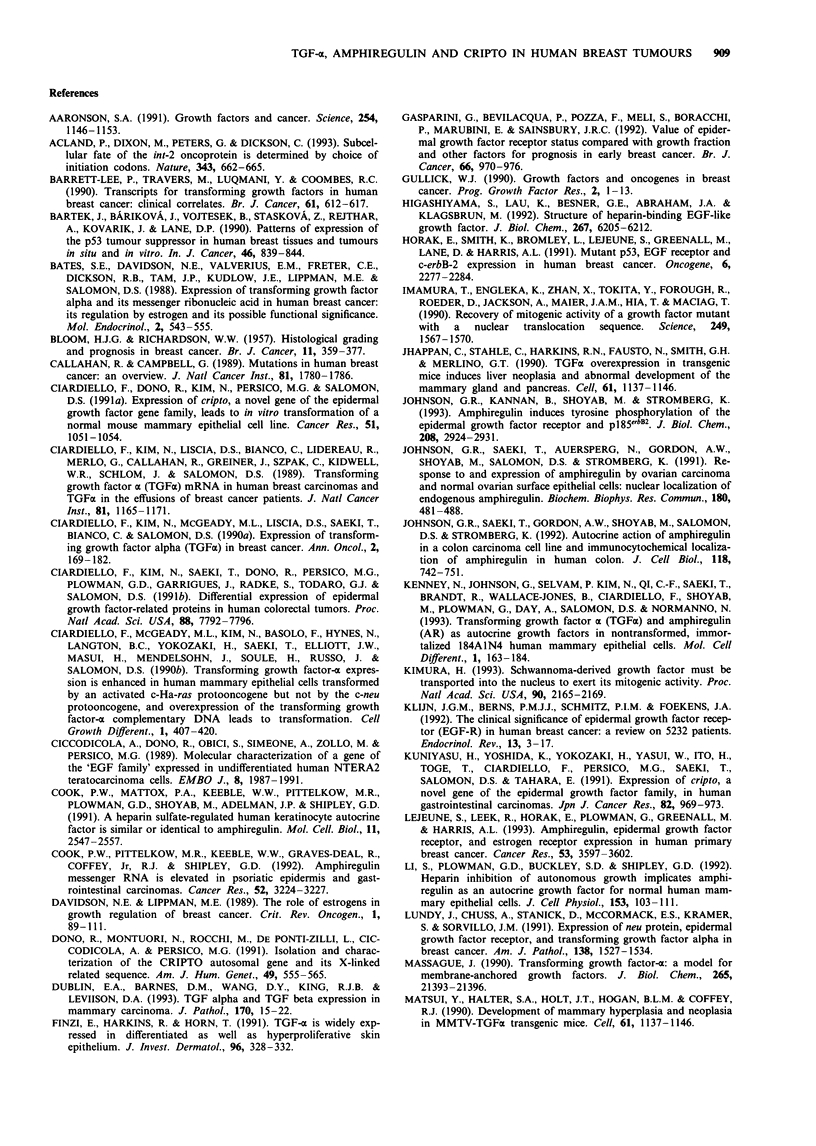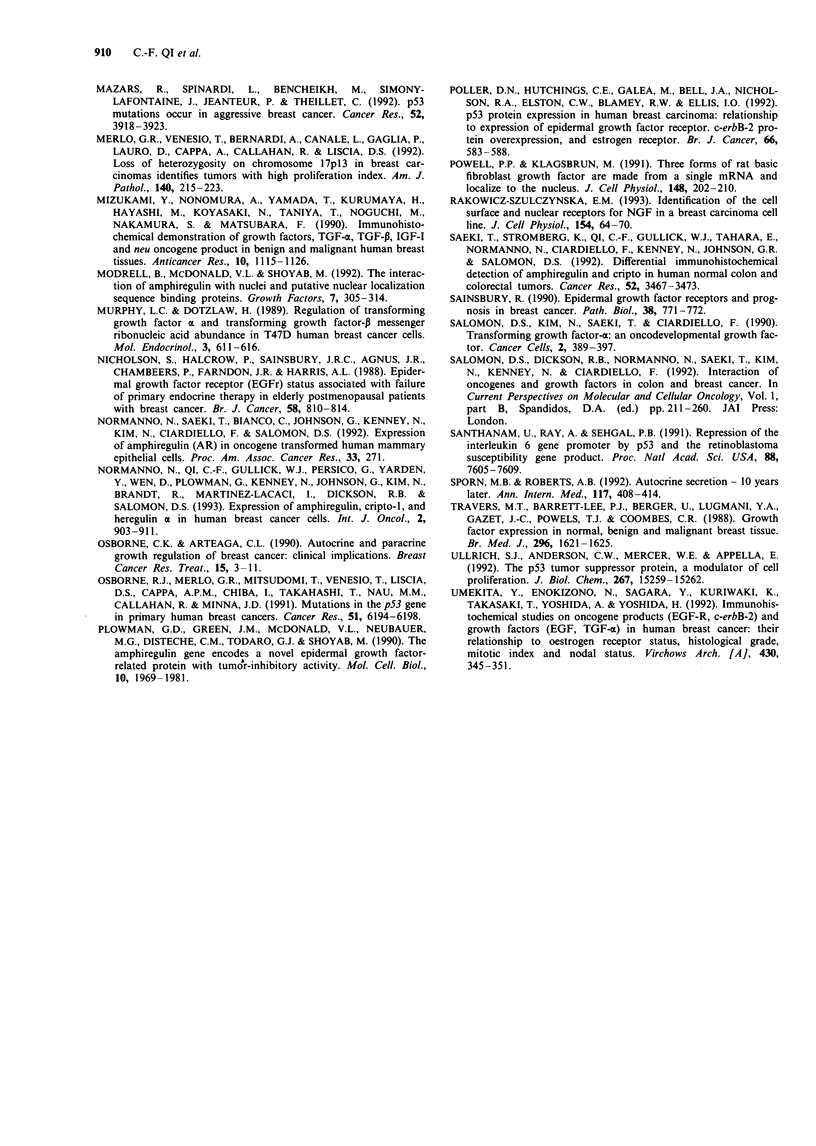# Expression of transforming growth factor alpha, amphiregulin and cripto-1 in human breast carcinomas.

**DOI:** 10.1038/bjc.1994.174

**Published:** 1994-05

**Authors:** C. F. Qi, D. S. Liscia, N. Normanno, G. Merlo, G. R. Johnson, W. J. Gullick, F. Ciardiello, T. Saeki, R. Brandt, N. Kim

**Affiliations:** Tumor Growth Factor Section, National Cancer Institute, National Institutes of Health, Bethesda, MD 20892.

## Abstract

**Images:**


Br. J. Cancer (1994), 69, 903-910
© Macmillan Press Ltd., 1994

### Expression of transforming growth factor α, amphiregulin and cripto-1 in human breast carcinomas

C.-F. Qi[1], D.S. Liscia[2], N. Normanno[1], G. Merlo[2], G.R. Johnson[3], W.J. Gullick[4], F. Ciardiello[5], T. Saeki[6], R. Brandt[1], N. Kim[1], N. Kenney[1] & D.S. Salomon[1]

[1]*Tumor Growth Factor Section, Laboratory of Tumor Immunology and Biology, Division of Cancer Biology, Diagnosis, and Centers, National Cancer Institute, Bethesda, Maryland 20892, USA;* [2]*Ospedale San Giovanni Vecchio, Servizio Anatomia Patologica, USL-1, Via Cavour 31, 10123 Turin, Italy;* [3]*Division of Cytokine Biology, Food and Drug Administration, Bethesda, Maryland 20892, USA;* [4]*Molecular Oncology Laboratory, Imperial Cancer Research Fund, Hammersmith Hospital, London, W12 0HS, UK;* [5]*Cattedra di Oncologia Medica, II Facolta' di Medicina e Chirurgia Universita' degli Studi di Napoli, 80131 Naples, Italy;* [6]*National Shikoku Cancer Center Hospital, Matsuyama, Japan.*

**Summary** The expression of three epidermal growth factor (EGF)-related peptides, transforming growth factor α (TGF-α), amphiregulin (AR) and cripto-1 (CR-1), was examined by immunocytochemistry (ICC) in 68 primary infiltrating ductal (IDCs) and infiltrating lobular breast carcinomas (ILCs), and in 23 adjacent non-involved human mammary tissue samples. Within the 68 IDC and ILC specimens, 54 (79%) expressed immunoreactive TGF-α, 52 (77%) expressed AR and 56 (82%) expressed CR-1. Cytoplasmic staining was observed with all of the antibodies, and this staining could be eliminated by preabsorption of the antibodies with the appropriate peptide immunogen. Cytoplasmic staining with all of the antibodies was confined to the carcinoma cells, since no specific immunoreactivity could be detected in the surrounding stromal or endothelial cells. In addition to cytoplasmic reactivity, the AR antibody also exhibited nuclear staining in a number of the carcinoma specimens. No significant correlations were found between the percentage of carcinoma cells that were positive for TGF-α, AR or CR-1 and oestrogen receptor status, axillary lymph node involvement, histological grade, tumour size, proliferative index, loss of heterozygosity on chromosome 17p or overall patient survival. However, a highly significant inverse correlation was observed between the average percentage of carcinoma cells that expressed AR in individual tumours and the presence of a point-mutated p53 gene. Likewise, a significantly higher percentage of tumour cells in the ILC group expressed AR as compared with the average percentage of tumour cells that expressed AR in the IDC group. Of the 23 adjacent, non-involved breast tissue samples, CR-1 could be detected by ICC in only three (13%), while TGF-α was found in six (26%) and AR in ten (43%) of the non-involved breast tissues. These data demonstrate that breast carcinomas express multiple EGF-related peptides and show that the differential expression of CR-1 in malignant breast epithelial cells may serve as a potential tumour marker for breast cancer.

A number of different growth factors have been demonstrated to be synthesised by normal and malignant mammary epithelial cells (Davidson & Lippman, 1989; Salomon *et al.*, 1992). These locally acting peptides may be important in regulating the growth of mammary epithelial cells through potential autocrine, juxtacrine and/or paracrine pathways (Aaronson, 1991; Sporn & Roberts, 1992). Epidermal growth factor (EGF) and peptides that are structurally related to EGF, such as transforming growth factor α (TGF-α), are potent mitogens for mammary epithelial cells (Ciardiello *et al.*, 1990*a*; Osborne & Arteaga, 1990). These peptides bind to the EGF receptor and activate its intrinsic tyrosine kinase activity (Massague, 1990; Salomon *et al.*, 1990). TGF-α has been detected in approximately 40–70% of primary human breast carcinomas, whereas EGF receptor expression occurs in nearly 50% of human breast tumours (Bates *et al.*, 1988; Travers *et al.*, 1988; Barrett-Lee *et al.*, 1990; Ciardiello *et al.*, 1990*a*; Klijn *et al.*, 1992; Dublin *et al.*, 1993). Coexpression of TGF-α and EGF receptor occurs in a majority of those breast tumours that are expressing either protein, suggesting that a potential autocrine or juxtacrine loop may exist *in vivo* in a subset of human breast tumours (Bates *et al.*, 1988; Travers *et al.*, 1988; Barrett-Lee *et al.*, 1990). EGF receptor status is also an important independent prognostic factor in human breast cancer (Sainsbury, 1990; Klijn *et al.*, 1992). High levels of EGF receptor expression are generally associated with tumours that have higher proliferative rates, with axillary lymph node involvement and with low disease-free or overall survival (Nicholson *et al.*, 1988; Sainsbury, 1990; Gasparini *et al.*, 1992). The activation of different proto-oncogenes and/or loss of expression of specific tumour-suppressor genes also frequently occurs in primary human breast tumours (Callahan & Campbell, 1989). These genetic alterations may affect the expression of and/or response to growth factors and can have a negative impact on overall patient survival (Aaronson, 1991; Salomon *et al.*, 1992). In this respect, inactivation of the p53 tumour-suppressor gene on chromosome 17p by point mutations and/or loss of heterozygosity (LOH) occurs in approximately 30–50% of primary human breast carcinomas (Callahan & Campbell, 1989; Bártek *et al.*, 1990; Horak *et al.*, 1991; Osborne *et al.*, 1991; Mazars *et al.*, 1992; Poller *et al.*, 1992). There is evidence demonstrating that the wild-type p53 gene encodes a nuclear phosphoprotein that functions as a transcription factor which can negatively regulate cell proliferation and which is involved in the pathway for other growth factor-controlled, cell cycle-related genes (Ullrich *et al.*, 1992).

TGF-α is only one of several proteins that can bind to the EGF receptor (Massague, 1990; Salomon *et al.*, 1990). Other newly discovered members of the EGF/TGF-α family of proteins include heparin-binding EGF-like growth factor (HB-EGF), amphiregulin (AR) and cripto-1 (CR-1, or teratocarcinoma-derived growth factor 1) (Ciccodicola *et al.*, 1989; Plowman *et al.*, 1990; Higashiyama *et al.*, 1992). AR is a 78 or 84 amino acid glycosylated protein that is initially synthesised as a 252 amino acid transmembrane precursor (Plowman *et al.*, 1990). Unlike EGF or TGF-α but similar to HB-EGF (Higashiyama *et al.*, 1992), AR can bind to heparin and has a hydrophilic 43 amino acid amino-terminal extension that contains at least two presumptive motifs which are similar to the nuclear localisation sequences associated with DNA-binding proteins (Plowman *et al.*, 1990). AR can bind to the EGF receptor, but with a lower affinity than EGF, can

Correspondence: D.S. Salomon, Tumor Growth Factor Section, Laboratory of Tumor Immunology and Biology, Building 10, Room 5B39, National Cancer Institute, National Institutes of Health, Bethesda, MD 20892, USA.
Received 9 July 1993; and in revised form 9 December 1993.

activate the EGF receptor tyrosine kinase and can transactivate the c-*erb*B-2 tyrosine kinase in several human breast and ovarian epithelial cell lines (Plowman *et al.*, 1990; Johnson *et al.*, 1993). Exogenous AR can either stimulate or inhibit the growth of different types of normal and malignant human epithelial cells, depending upon the concentration, presence of other growth factors and nature of the target cell (Plowman *et al.*, 1990; Johnson *et al.*, 1991, 1992; Normanno *et al.*, 1992; Li *et al.*, 1992). AR is expressed in and is able to function as an autocrine growth factor for several human mammary epithelial cell strains and for c-Ha-*ras*- and c-*erb*B-2-transformed MCF-10A human mammary epithelial cells (Li *et al.*, 1992; Normanno *et al.*, 1992). CR-1 is a 188 amino acid protein that, unlike other members of the EGF/TGF-α family, lacks a hydrophobic signal peptide and transmembrane domain, but which contains a central region of approximately 37 amino acids that shares structural homology with peptides within this family (Ciccodicola *et al.*, 1989; Dono *et al.*, 1991). Although a recombinant or naturally occurring CR-1 protein has not yet been obtained to ascertain its biological properties, overexpression of the human *CR-1* gene can lead to the *in vitro* transformation of mouse NIH3T3 fibroblasts or mouse NOG-8 mammary epithelial cells, demonstrating that CR-1, like TGF-α and AR, can function as an autocrine growth factor and/or dominantly transforming oncogene (Ciccodicola *et al.*, 1989; Ciardiello *et al.*, 1990*b*, 1991*a*; Jhappan *et al.*, 1990; Matsui *et al.*, 1990). In addition, CR-1 is expressed in a majority of human colorectal and gastric carcinoma cell lines and tumours (Ciccodicola *et al.*, 1991*b*; Kuniyasu *et al.*, 1991; Saeki *et al.*, 1992).

TGF-α, AR and CR-1 mRNA transcripts are expressed in a number of different human breast cancer cell lines (Bates *et al.*, 1988; Davidson & Lippman, 1989; Murphy & Dotzlaw, 1989; Plowman *et al.*, 1990; Normanno *et al.*, 1993). Since there is little or no information on the frequency and level of expression of AR or CR-1 in primary human breast lesions, we have analysed a small panel of infiltrating human breast carcinomas and non-involved breast tissues adjacent to carcinomas for TGF-α, AR and CR-1 expression. Immunocytochemistry (ICC) using peptide-specific polyclonal antibodies that are capable of detecting these proteins in formalin-fixed, paraffin-embedded tissues was used to ascertain if these peptides can be localised in mammary epithelial cells and to determine if there is any differential expression of these proteins between non-involved and malignant breast tissues.

### Materials and methods

### *Human breast tissues*

Paraffin blocks of formalin-fixed tissue and frozen tissue samples that were obtained from 68 primary infiltrating breast carcinomas with 23 cases of non-involved breast tissue were collected at the S. Giovanni Vecchio Hospital, Turin, Italy. A portion of each tumour specimen was frozen in liquid nitrogen at −70°C until extraction of genomic DNA or before evaluation of the labelling index by bromodeoxyuridine (BrdU) incorporation. Patients were graded histopathologically according to the Bloom and Richardson (1957) method and by the UICC TNM (tumour, nodes, metastases) staging system.

### *Polyclonal antibodies*

Rabbit antibody R9 was generated against recombinant human TGF-α that had been conjugated to keyhole limpet haemocyanin (KLH) as previously described (Finzi *et al.*, 1991). The anti-AR antibody (AR-Ab-1) was raised against a 19-mer synthetic peptide that corresponds to residues 8–26 in the human AR protein as previously described (Johnson *et al.*, 1991, 1992). The specificity and reactivity of the affinity-purified AR-Ab-1 immunoglobulin G (IgG) was evaluated as previously described (Johnson *et al.*, 1991, 1992). The AR-Ab-1 antibody can detect recombinant human AR in an enzyme-linked immunosorbent assay (ELISA). In addition, the AR-Ab-1 IgG is able to detect a specific $M_r$ 18,000–25,000 glycoprotein in the conditioned medium obtained from 12-*O*-tetradecanoylphorbol-13-acetate (TPA)-treated MCF-7 human breast cancer cells and does not react with either EGF or TGF-α (Johnson *et al.*, 1991). The anti-CR-1 antibody (CR-1 Ab) was generated against a 17-mer synthetic peptide that corresponds to amino residues 97–113 in the human CR-1 protein and that represents the carboxy terminus of the 37 amino acid EGF-like region as previously described (Saeki *et al.*, 1992). The CR-1 Ab is able to detect an $M_r$ 32,000 MS2–CR-1 fusion protein derived from *Escherichia coli*, an $M_r$ 28,000 thrombomodulin signal peptide CR-1 protein derived from human CR-1-transfected CHO cells and a specific $M_r$ 36,000 endogenous CR-1 protein in several human tumour cell lines that express the 2.2 kb CR-1 mRNA following Western blot analysis (Saeki *et al.*, 1992; R. Brandt *et al.*, in preparation). CR-1 Ab does not recognise either human TGF-α or AR in an ELISA, but reacts strongly with the 17-mer CR-1 peptide immunogen.

### *Immunocytochemistry and evaluation of immunoperoxidase staining*

Paraffin-embedded tissue sections (5 μm) were deparaffinised in xylene and rehydrated in a graded series of ethanol. The slides were then treated for 30 min at 20°C with methanol containing 0.3% hydrogen peroxide to block any endogenous peroxidase activity. After several washes with phosphate-buffered saline (PBS), the sections were incubated for 45 min with 10% goat serum, washed with PBS and incubated for 12 h with the appropriate primary antibody at 4°C. Sections were then washed three times with PBS and treated with secondary biotinylated goat anti-rabbit IgG (1:200 dilution, Vectastain AC kit; Vector Laboratory, Burlingame, CA, USA) for 30 min. Following several washes with PBS, the slides were reacted for 30 min with avidin dehydrogenase-biotinylated horseradish peroxidase H complex, rinsed twice in PBS and incubated for 2 min in 0.05% diaminobenzidine and in 0.01% hydrogen peroxide. The slides were then rinsed in distilled water, counterstained with haematoxylin and mounted. The anti-TGF-α R9 primary antibody was utilised at a 1:200 dilution. In some cases, the R9 antibody was preabsorbed with 10 $\mu g\ ml^{-1}$ human TGF-α (Bachem, Torrance, CA, USA) for 2 h at 37°C. The AR-Ab-1 IgG was used at 10 $\mu g\ ml^{-1}$, which in some instances was preabsorbed with 20 $\mu g\ ml^{-1}$ 19-mer AR synthetic peptide for 2 h at 37°C. The CR-1 Ab was utilised at a 1:400 dilution and, in some cases, was preabsorbed with 20 $\mu g\ ml^{-1}$ 17-mer CR-1 synthetic peptide for 2 h at 37°C. Slides were graded for staining specificity and intensity and for the percentage of immunopositive cells as previously described (Johnson *et al.*, 1991; Saeki *et al.*, 1992). Non-specific staining was evaluated for each specimen using either a similar concentration or dilution of preimmune rabbit serum or IgG, or by absorbing the primary antibody with the appropriate peptide immunogen. The number of immunopositive cells per slide was stratified into three groups based upon the percentage of positive cells: group 1, $<30\%$; group 2, 30–60%; and group 3, $>60\%$.

For analysing proliferation in tumour specimens, frozen 5 μm sections which were obtained in parallel with the formalin-fixed sections were incubated with a 1:50 dilution of Ki-67 monoclonal antibody (Dakopatts, UK) for 60 min. The slides were then washed several times with PBS, reacted with a 1:200 dilution of biotinylated horse anti-mouse IgG for 30 min, washed with PBS and stained with avidin dehydrogenase–biotinylated horseradish peroxidase H complex for 30 min. Nuclei that were stained with the Ki-67 antibody were counted in each specimen and quantitated as a fraction of the total number of cells in each sample. Approximately 500 tumour cells were screened for each specimen, and values were expressed as a percentage of positively stained nuclei. A panel of 700 breast tumours was used to determine the median cut-off point, 9.0%, for Ki-67 nuclear staining. Using this value, tumours were classified in either a high or low index rank.

*Oestrogen receptor (ER) assay*

Cytosolic ER was assayed by the dextran-coated charcoal (DCC) method as previously described (Merlo *et al.*, 1992).

*Southern blot analysis and DNA probes*

High molecular weight DNA was extracted and blotted as previously described (Merlo *et al.*, 1992). The DNA was immobilised by UV cross-linking followed by prehybridisation and hybridisation with the following $^{32}$P-labelled DNA probes: pYNZ22.1/D17S30 marker probe (ATCC no. 57575 probe, Rockville, MD, USA) (Osborne *et al.*, 1991; Merlo *et al.*, 1992) and the probe p144D6 (Merlo *et al.*, 1992).

*Single-strand conformation polymorphism (SSCP) analysis of genomic and cDNA for p53 point mutations*

The polymerase chain reaction (PCR)/SSCP method was modified to screen for point mutations in the p53 gene as previously described (Osborne *et al.*, 1991). Genomic DNA/PCR fragments of 438 bp spanning exons 5 and 6 or 670 bp containing exons 7 and 8, and cDNA/PCR fragments spanning exons 4–7 (codons 242–327) or exons 7–9 (codons 242–327) were amplified using 100 ng of genomic DNA or 250 ng of random-primed cDNA as templates and 0.5 µl of [$^{32}$P]dCTP (Amersham, Arlington Heights, IL, USA) in 10 ml reaction volumes. To localise possible point mutations to a specific exon, 1 µl of the PCR product was digested with *Aat*I (USB, Cleveland, OH, USA) for the exon 5/6 fragment, or *Dra*I (Bethesda Research Laboratory, Gaithersburg, MD, USA) for the exon 7/8, and *Alw*NI (New England Biolabs, Beverly, MA, USA) for the cDNA/PCR product. The reaction was diluted 1:5 with loading buffer (95% formamide, 2 mM EDTA, pH 8.3). Two microlitres of each diluted sample was denatured (90°C for 5 min) and loaded onto a 6% non-denaturing acrylamide gel in 89 mM Tris-borate, 2 mM EDTA, pH 8.3, and electrophoresed for 5 h at 4°C at 25 W. The gels were then dried and exposed to X-ray film.

*BrdU incorporation*

In addition to monitoring for Ki-67 labelling of nuclei, the proliferation index was also assessed by measuring the levels of incorporation of BrdU (0.1 mM) into fresh tumour fragments from each specimen after a 3 h incubation in short-term tissue culture as previously described (Merlo *et al.*, 1992). Approximately 500 tumour cells were screened for each specimen and values were expressed as a percentage of positively stained nuclei. A panel of 700 breast tumours was used to determine the median cut-off point, 7.0%, for BrdU incorporation. Using this value, tumours were classified in either a high or low proliferation index rank.

*Statistical analysis*

The tests of significance were the Wilcoxon rank sum test and the Kruskal–Wallis test for non-parametric analysis of variance. These tests were utilised to compare the average percentage of tumour cells in any given specimen that were positive for TGF-α, AR or CR-1 with tumour size (T1, T2 and T3), axillary lymph node status (N0, axillary node negative; N1, axillary node positive), histological grade (G1, G2 and G3), histology of the tumour (IDC, infiltrating ductal carcinoma; ILC, infiltrating lobular carcinoma), ER and PR status, BrdU rank, Ki-67 rank and loss of heterozygosity (LOH) on chromosome 17p, which were assessed in 65 out of 68 cases, and the presence or absence of p53 mutations, which was assessed in 56 cases. The same comparison Pearson's regression coefficient test was used to assess possible linear correlations between TGF-α, AR or CR-1 staining with BrdU, Ki-67, ER status and patient age.

### Results

TGF-α mRNA has previously been identified in approximately 40–70% of infiltrating breast carcinomas (Bates *et al.*, 1988; Travers *et al.*, 1988; Ciardiello *et al.*, 1989; Barrett-Lee *et al.*, 1990). To ascertain if there is an equivalent frequency of TGF-α protein expression in breast lesions and to determine if there are any differences in the frequency of TGF-α protein expression between non-malignant and malignant breast tissues, we examined, by ICC, 68 infiltrating breast carcinomas and 23 non-involved breast tissues that were adjacent to carcinomas. Of the 68 primary human breast tumours, 54 (79%) were stained with the R9 rabbit anti-TGF-α antibody (Table I). Immunoreactivity with the R9-TGF-α antibody was generally confined to the carcinoma cells, since there was very little staining of the surrounding stroma, smooth muscle or capillary endothelial cells (Figure 1b). Staining was cytoplasmic with some cell membrane reactivity. This staining pattern was specific, since no staining was detected in parallel sections that had been incubated either with a similar dilution of preimmune rabbit serum (data not shown) or with R9 antiserum that had been preabsorbed with human TGF-α (Figure 1a). Heterogeneity of TGF-α expression was observed in the carcinoma population. In eight (12%) tumours over 60% of the carcinoma cells were stained with R9 anti-TGF-α antibody, while in 18 (26%) tumours 30–60% stained with the antibody and in 28 (41%) less than 30% of the tumour cells expressed immunoreactive TGF-α (Table I). In contrast to the relatively high frequency of TGF-α expression in the carcinomas, only 6 (23%) of the 23 non-involved breast tissues that were adjacent to carcinomas exhibited specific reactivity with the R9 antibody (Figure 1c). In addition, staining in the non-involved breast tissue was generally less intense than in the adjacent tumour cells. Similar to the breast carcinoma cells, staining was cytoplasmic and was in most instances restricted to ductal epithelial cells.

The relative distribution and frequency of CR-1 expression

**Table I** Expression of immunoreactive AR, TGF-α and CR-1 in human breast tissues

| | *AR* | *TGF-α* | *CR-1* |
|---|---|---|---|
| Mammary epithelium adjacent to carcinoma | 10/23 (43%) | 6/23 (26%) | 3/23 (13%) |
| Breast carcinomas | 52/68 (77%) | 54/68 (79%) | 56/68 (82%) |
| Positive tumour cells | | | |
| Over 60% | 9/68 (13%) | 8/68 (12%) | 21/68 (31%) |
| Between 30% and 60% | 16/68 (24%) | 18/68 (26%) | 17/68 (25%) |
| Less than 30% | 27/68 (40%) | 28/68 (41%) | 18/68 (26%) |
| Negative | 16/68 (23%) | 14/68 (21%) | 12/68 (18%) |

Numbers in parentheses are the percentages of total that were immunopositive for each protein.

**Figure 1** Immunoperoxidase staining of formalin-fixed paraffin-embedded breast tissues using anti-TGF-α R9 antibody (**a**–**c**), anti-CR-1 antibody (**d**–**f**) or anti AR-Ab-1 (**g**–**i**). In **a**, **d** and **g**, breast carcinoma specimens were reacted with antiserum that had been preabsorbed with either recombinant TGF-α (**a**), the 17-mer CR-1 synthetic peptide (**d**), or with the 19-mer AR synthetic peptide (**g**), × 400 (**a**, **d**, **g**); in **b**, **e** and **h**, serial breast carcinoma sections were reacted with a 1:200 dilution of the R9 antibody (**b**), a 1:400 dilution of the CR-1 antibody (**e**) or with 10 $\mu g\ ml^{-1}$ AR-Ab-1 IgG (**h**), × 400 (**b**, **e**, **h**); in **c**, **f** and **i**, adjacent non-involved breast epithelium was reacted with a 1:200 dilution of the R9 antibody (**c**), a 1:400 dilution of the CR-1 antibody (**f**) or with 10 $\mu g\ ml^{-1}$ AR-Ab-1 IgG (**i**), × 200 (**f**, **i**) or × 400 (**c**).

in breast tissues was similar to TGF-α (Table I). Of the 68 breast carcinomas, 56 (82%) exhibited intense cytoplasmic and perinuclear staining with the cripto anti-CR-1 antibody (Figure 1e), while no staining was observed in tumour sections after preabsorption of the CR-1 antibody with the 17-mer peptide immunogen (Figure 1d). In addition, very little specific immunoperoxidase staining with the anti-CR-1 antibody was observed within the stroma or within the vascular elements of the carcinomas. As was the case with TGF-α expression, a marked heterogeneity was observed in the percentage of carcinoma cells that were stained with the CR-1 antibody within any one specimen. A greater proportion of the carcinomas expressed CR-1 as compared with TGF-α: in 21 (31%) carcinomas over 60% of the tumour cells stained with the CR-1 antibody, whereas in 17 (25%) of the carinomas in this CR-1-positive group 30–60% of the cells stained and in 18 (26%) carcinomas less than 30% of the tumour cells expressed CR-1 (Table I). The frequency of CR-1 expression in adjacent non-involved breast tissue was lower than TGF-α expression. Only 3 (13%) of the 23 adjacent breast tissues exhibited staining with the CR-1 antibody. However, in the three specimens that were positive, only a very weak staining of the ductal epithelial cells was observed (Figure 1f).

AR expression in the breast carcinomas was then evaluated since a specific 1.4 kb mRNA transcript for this growth factor has recently been shown to be expressed in several normal human mammary epithelial cell strains and in several human breast cancer cell lines (Plowman *et al.*, 1990; Cook *et al.*, 1991; Li *et al.*, 1992; Normanno *et al.*, 1992, 1993). In the 68 breast tumours, 52 (77%) showed specific staining with the anti-AR Ab-1 antibody (Table I). Specific staining was confined to the carcinoma cells and was both cytoplasmic and nuclear (Figure 1h). Preabsorption of the anti-AR IgG fraction with the 19-mer synthetic peptide immunogen abolished the staining (Figure 1g). In 13% of the tumours, 60% or more of the carcinoma cells within a given specimen were stained with the AR-Ab-1 antibody, whereas in 24% of the carcinomas 30–60% of the tumour cells stained, and in 40% of the tumours less than 30% of the carcinoma cells expressed AR. The frequency of AR expression in adjacent non-involved breast tissue was higher than either TGF-α or CR-1 expression. Of the 23 adjacent breast tissues, ten (43%) exhibited staining with the anti-AR Ab-1 antibody. In non-involved breast tissues that were positive for AR, less intense cytoplasmic staining of the ductal epithelial cells was generally observed as compared with the intensity of staining in the carcinoma cells (Figure 1i). A majority of the breast carcinomas expressed two or more of these proteins, since 45 (66%) of the 68 breast carcinomas were immunopositive with all three antibodes, whereas only 4 (6%) of the 68 breast carcinomas were negative for all three proteins.

To ascertain if the frequency of expression of TGF-α, CR-1 or AR in the infiltrating breast carcinomas might correlate with any pathological indicators of prognosis, the percentages of tumour cells that were ICC positive for either TGF-α, CR-1 or AR within individual carcinoma specimens were statistically compared by the Wilcoxon rank sum test with tumour size (T), axillary lymph node involvement (N), histological grade (G), histological type (IDC *vs* ILC), ER receptor status, Ki-67 nuclear staining and BrdU incorporation as an index of cell proliferation, LOH on chromosome 17p and the presence of point mutations within the p53 gene (Table II). A variable number of tumours were present within

**Table II** Association of average percentage of breast carcinoma cells that are positive for AR, TGF-α and CR-1 with clinicopathological parameters

| | | *AR* | | *TGF-α* | | *CR-1* |
|---|---|---|---|---|---|---|
| *Parameter* | *n* | *Mean (%)* | *n* | *Mean (%)* | *n* | *Mean (%)* |
| BrdU low | 38 | 30.2 | 38 | 30.5 | 38 | 42.8 |
| | | − 0.64[a] | | − 0.90 | | − 0.72 |
| BrdU high | 27 | 23.7 | 27 | 30.7 | 27 | 40.7 |
| Ki67 low | 34 | 25.1 | 34 | 27.0 | 34 | 40.3 |
| | | − 0.32 | | − 0.26 | | − 0.71 |
| Ki67 high | 31 | 30.1 | 31 | 34.5 | 31 | 43.8 |
| P53 normal | 40 | 31.6 | 40 | 35.0 | 40 | 45.5 |
| | | − 0.008[b] | | − 0.08 | | − 0.19 |
| P53 mutant | 16 | 11.5 | 16 | 21.8 | 16 | 34.3 |
| P17 normal | 39 | 24.6 | 39 | 31.0 | 39 | 38.4 |
| | | − 0.10 | | − 0.96 | | − 0.26 |
| P17 LOH | 26 | 31.9 | 26 | 30.0 | 26 | 47.3 |
| Grade 1 | 1 | 60.0 | 1 | 90.0 | 1 | 50.0 |
| Grade 2 | 36 | 24.8 | 36 | 30.5 | 36 | 42.7 |
| | | − 0.43 | | − 0.25 | | − 0.89 |
| Grade 3 | 19 | 22.3 | 19 | 31.5 | 19 | 47.3 |
| N0 | 27 | 29.8 | 27 | 29.2 | 27 | 38.1 |
| | | − 0.45 | | − 0.62 | | − 0.34 |
| N1 | 38 | 25.9 | 38 | 31.5 | 38 | 44.7 |
| T1 | 25 | 33.0 | 25 | 35.2 | 25 | 43.2 |
| T2 | 35 | 25.0 | 35 | 28.2 | 35 | 44.8 |
| | | − 0.57 | | − 0.52 | | − 0.13 |
| T3 | 5 | 18.0 | 5 | 24.0 | 5 | 16.0 |
| ER negative | 41 | 26.8 | 41 | 30.4 | 41 | 39.7 |
| | | − 0.95 | | − 0.85 | | − 0.41 |
| ER positive | 24 | 28.7 | 24 | 30.8 | 24 | 45.8 |
| IDC | 56 | 24.6 | 56 | 31.9 | 56 | 44.4 |
| | | − 0.01[b] | | − 0.41 | | − 0.10 |
| ILC | 9 | 45.5 | 9 | 22.2 | 9 | 26.6 |

[a]*P*-values determined by Wilcoxon rank sum test. [b]Statistically significant.

each group since several of these parameters were not evaluated for all 68 of the tumour samples. Histologically, a majority of the carcinomas, 56 out of 65 (86%), that were analysed for these prognostic indicators were IDCs, while the remaining nine (14%) were ILCs. There was no statistically significant association between the IDC and ILC groups with respect to the mean number of carcinoma cells that were immunopositive for either TGF-α or CR-1. However, a statistically significantly higher percentage of carcinoma cells expressed AR in the ILC group than in the IDC group (46% *vs* 25%; $P = 0.01$). With the exception of p53 status and histology, there was no significant association between the other prognostic factors and the average percentage of tumour cells within any one specimen that were expressing any of these three EGF-related proteins (Table II).

The p53 gene maps to chromosome 17p and allelic loss and/or mutations within exons 4–9 of the gene occur in nearly 30–50% of breast tumours (Bartek *et al*., 1990; Osborne *et al*., 1991; Mazars *et al*., 1992). SSCP analysis for the presence of p53 point mutations within exons 4–9 or enhanced immunostaining with the anti-p53 antibodies yielded similar results. SSCP analysis demonstrated that 16 (29%) of the 56 breast tumours that were assessed had one or more point mutations. More importantly, there was a statistically significant inverse association between tumours that had a higher average percentage of tumour cells that expressed AR and the presence of a p53 mutation(s) (32% *vs* 12%; $P = 0.008$). This relationship with p53 mutations was not observed for TGF-α or CR-1 expression.

### Discussion

Abnormal expression of growth factors and their cognate receptors has been implicated in the pathogenesis of a number of different types of malignancies (Davidson & Lippman, 1989; Ciardiello *et al*., 1990*a*; Gullick, 1990; Osborne & Arteaga, 1990; Salomon *et al*., 1990, 1992; Aaronson, 1991; Sporn & Roberts, 1992). A functional connection exists between oncogenes and growth factors since some proto-oncogenes have the capacity to code for growth factors or growth factor receptors (Aaronson, 1991; Sporn & Roberts, 1992). In this regard, overexpression of the EGF receptor (c-*erb*B) coupled with the enhanced production of ligands that activate this receptor such as TGF-α have been found in a number of human tumour cell lines and in several different types of primary human carcinomas, including breast carcinomas, suggesting that a possible autocrine, juxtacrine and/or paracrine mechanism of growth regulation involving this receptor signalling pathway might be operative *in vitro* and *in vivo* (Bates *et al*., 1988; Travers *et al*., 1988; Ciardiello *et al*., 1989, 1990*a*; Barrett-Lee *et al*., 1990; Massague, 1990; Osborne & Arteaga, 1990; Salomon *et al*., 1990; Aaronson, 1991; Sporn & Roberts, 1992).

The present study is the first to demonstrate by ICC that not only TGF-α but also two recently discovered members of the EGF/TGF-α family of growth factors, AR and CR-1, are expressed at a high frequency and in some cases preferentially in a majority of primary human infiltrating ductal and lobular breast carcinomas. TGF-α was expressed in 79% of a small cohort of 68 human primary breast carcinomas that were examined. Staining was restricted to the cytoplasm and cell membranes of the tumour epithelial cells. Lundy *et al*. (1991) reported TGF-α expression by ICC in 68% of 51 breast carcinomas, while two additional studies have reported 28% and 50% of tumours showing staining for immunoreactive TGF-α protein (Mizukami *et al*., 1990; Umekita *et al*., 1992). This difference in the frequency of TGF-α protein expression might be due to the use of monoclonal anti-TGF-α antibodies in these studies as compared with the utilisation of a polyclonal anti-TGF-α antibody in the present study. Formalin fixation might mask specific epitopes in tissues that monoclonal antibodies would normally recognise, whereas the polyclonal antibody might be less sensitive to such changes since multiple epitopes are usually being detected. In contrast to the high frequency of TGF-α expression in breast carcinomas, low levels of TGF-α protein were found in breast epithelium in that only 26% of adjacent non-involved mammary epithelium stained positively for TGF-α, and generally at a lower level of intensity than the corresponding carcinomas. This finding is in agreement with those of Barrett-Lee *et al*. (1990), who found low levels of expression of TGF-α mRNA in 33% (two out of six) of normal human breast specimens, and with the results of Mizukami *et al*. (1990), in which there was little immunoreactive TGF-α in ten normal breast tissue specimens. In the present study, there was no significant correlation between the average percentage of carcinoma cells in any given carcinoma specimen that were expressing TGF-α and other clinical parameters. A similar lack of association between TGF-α mRNA expression and other clinical and pathophysiological parameters has been noted in several previous studies (Bates *et al*., 1988; Travers *et al*., 1988; Ciardiello *et al*., 1989; Barrett-Lee *et al*., 1990).

AR is a newly discovered EGF-related growth factor that is expressed in normal mammary and ovarian epithelial cells (Cook *et al*., 1991; Johnson *et al*., 1991; Li *et al*., 1992; Kenney *et al*., 1993), in several human ovarian, colon and breast carcinoma cell lines (Ciardiello *et al*., 1991*b*; Johnson *et al*., 1991, 1992; Cook *et al*., 1992; Saeki *et al*., 1992; Normanno *et al*., 1993) and in a majority of primary human colorectal carcinomas (Ciardiello *et al*., 1991*b*; Johnson *et al*., 1992; Saeki *et al*., 1992). The present study is the first to demonstrate the presence of immunoreactive AR in a subset of primary human breast carcinomas. An enhanced level of AR expression was observed in 77% of the primary breast carcinomas that were examined in this study. Lower levels of expression of AR were detected in 43% of the breast epithelium specimens that were adjacent to carcinomas. Recently, Lejeune *et al*. (1993) have analysed a series of primary breast tumours for AR expression. They found that approximately 35% of the breast tumours expressed AR mRNA and AR protein by immunocytochemistry using an anti-AR mouse monoclonal antibody. In their study, there was no association between AR expression and several prognostic factors with the exception of lymph node status, expression being more common in lymph node-positive cases than in lymph node-negative cases. In the present study, involving both normal and malignant breast tissues, staining was restricted to the mammary epithelial cells, was in most cases cytoplasmic and was generally more frequent and intense in the carcinoma cells than in the surrounding non-involved breast epithelium. In addition, in the carcinoma cells, frequent nuclear staining was also observed in a number of the specimens. Immunolocalisation of AR in the nucleus has been noted in normal colonic and ovarian surface epithelial cells, in several human breast and colon cancer cell lines and in colon and ovarian carcinomas (Johnson *et al*., 1991, 1992; Saeki *et al*., 1992; Normanno *et al*., 1992, 1993). Nuclear localisation of this protein may be due to the presence of two consensus sequences in the amino-terminal region that could serve as potential nuclear targeting regions to translocate the peptide into the nucleus (Plowman *et al*., 1990; Modrell *et al*., 1992). In this respect, addition of exogenous [$^{125}$I]AR to human A431 epidermoid carcinoma cells or to human HTB-132 breast carcinoma cells leads to nuclear sequestration of the intact peptide and binding to two nuclear phosphoproteins (Modrell *et al*., 1992). This may not be unique to AR since other growth factors such as HB-EGF, nerve growth factor, interleukin 1, basic fibroblast growth factor (bFGF) and the *int*-2 FGF-related protein also possess nuclear localisation sequences and can be detected in the nucleus (Imamura *et al*., 1990; Powell & Klagsbrun, 1991; Higashiyama *et al*., 1992; Acland *et al*., 1993; Rakowicz-Szulczynska, 1993). In this regard, multiple forms of bFGF have been found in the nuclei of target cells, and deletion of the amino terminal nuclear retention sequences from either bFGF or from AR can abolish their mitogenic activity (Imamura *et al*., 1990; Powell & Klagsbrun, 1991; Kimura, 1993).

The inverse association between the average number of carcinoma cells that express AR and the p53 status of the breast tumour is intriguing. A significantly greater percentage of carcinoma cells in breast tumours that contained a normal p53 gene express AR than in tumour specimens that possessed p53 mutations. Inactivation of p53 tumour-suppressor activity in human breast tumours can occur by missense mutations in one allele, which is frequently followed by LOH or by a reduction to homozygosity in the second allele (Callahan & Campbell, 1989; Osborne *et al*., 1991; Mazars *et al*., 1992; Ullrich *et al*., 1992). It is possible that the wild-type p53 protein might positively regulate the expression of AR. In this regard, wild-type p53 can repress the promoter for interleukin 6, another autocrine growth factor (Santhanam *et al*., 1991). It is also of interest to note that there is a significant association in primary breast carcinomas between EGF receptor levels and the presence of p53 mutations, suggesting that a mutant p53 protein might up-regulate EGF receptor expression (Horak *et al*., 1991; Poller *et al*., 1992). Finally, expression of p53 mutations in one study was found to be rare in infiltrating lobular carcinomas (Poller *et al*., 1992), which may relate to the findings in the present study demonstrating that the average percentage of carcinoma cells that were expressing AR in individual tumours was significantly higher in infiltrating lobular carcinomas, which tend to be histologically more differentiated, than in infiltrating ductal carcinomas. A similar situation has been found in human primary colorectal tumours, in which a higher frequency of AR expression was detected in well-differentiated carcinomas than in poorly differentiated tumours (Saeki *et al*., 1992).

Expression of CR-1 has been detected in only a limited number of malignant cells, such as undifferentiated mouse and human embryonal carcinoma cells, several human gastric, colon and breast cancer cell lines and primary human colorectal and gastric carcinomas (Ciccodicola *et al*., 1989, 1991*b*; Kuniyasu *et al*., 1991; Saeki *et al*., 1992; Normanno *et al*., 1993). Eighty-two per cent of the breast carcinomas were immunopositive for CR-1, while only 13% of the adjacent non-involved breast epithelial specimens were reactive with the anti-CR-1 antibody. These values are very close to the frequency of CR-1 expression that was observed in colorectal tumours and in adjoining, non-involved colonic mucosa. The antibody preferentially stained the carcinoma cells and showed cytoplasmic reactivity in most cases. There was no significant correlation between the average number of carcinoma cells that exhibited CR-1 reactivity in any given carcinoma specimen and several clinical parameters that were examined. However, the greater differential expression of CR-1 in breast carcinomas relative to TGF-α and AR, which were generally expressed at a higher frequency than CR-1 in the non-involved breast epithelium, suggests that CR-1 may serve as a potential breast tumour marker. The significance of this observation is unclear since a recombinant protein is not available to determine whether the CR-1 protein has any biological activity. However, preliminary evidence using refolded peptides that correspond to the EGF-like domain of the CR-1 protein has demonstrated that these peptides are able to stimulate the proliferation of non-transformed 184A1N4 human mammary epithelial cells and several human breast cancer cell lines (Brandt *et al*., in preparation). Coexpression of TGF-α, AR and CR-1 occurred in 66% (45 out of 68) of the breast carcinomas, while only 6% (4 out of 68) of the tumours failed to express all three proteins. The significance of the high frequency of coexpression of three EGF-related peptides in breast carcinomas has yet to be clarified. However, this is apparently not unique to breast carcinomas, since a similar situation with respect to the coexpression of TGF-α, AR and CR-1 mRNA transcripts and coexpression of immunoreactive AR and CR-1 proteins is found in human colorectal carcinomas (Ciardiello *et al*., 1991*b*; Cook *et al*., 1992; Saeki *et al*., 1992). At least two of these three EGF-related peptides, TGF-α and AR, are bona fide growth factors that function exclusively through the EGF receptor (Massague, 1990; Plowman *et al*., 1990; Salomon *et al*., 1990; Johnson *et al*., 1993). The apparent redundancy in the expression in mammary epithelial cells of two structurally and biologically related peptides suggests that these peptides may be involved in the regulation of additional biological properties other than cell proliferation such as differentiation. This may be the case since in normal colon specimens immunoreactive AR protein is not found in the proliferative stem cell population of the crypts but is preferentially expressed in the terminally differentiated, non-proliferative columnar and secretory epithelial cells of the villous mucosa and is found at a higher frequency in better differentiated colon carcinomas (Johnson *et al*., 1992; Saeki *et al*., 1992).

The authors would like to express their gratitude to Dr Beatrice Langton, Berlex Biosciences, for generously providing the anti-TGF-α R9 antibody.

**Abbreviations:** EGF, epidermal growth factor; TGF-α, transforming growth factor α; AR, amphiregulin; HB-EGF, heparin-binding EGF-like growth factor; CR-1, cripto-1; ICC, immunocytochemistry; LOH, loss of heterozygosity; IgG, immunoglobulin G; KLH, keyhole limpet haemocyanin; kb, kilobase; RFLP, restriction fragment length polymorphism; SSCP, single-strand conformation polymorphism; cDNA, complementary DNA; PCR, polymerase chain reaction; BrdU, bromodeoxyuridine; DCC, dextran-coated charcoal; ER, estrogen receptor, PR, progesterone receptor; IDC, infiltrating ductal carcinoma; ILC, infiltrating lobular carcinoma; TNM, tumour, nodes, metastases.

### References

AARONSON, S.A. (1991). Growth factors and cancer. *Science*, **254**, 1146–1153.

ACLAND, P., DIXON, M., PETERS, G. & DICKSON, C. (1993). Subcellular fate of the *int*-2 oncoprotein is determined by choice of initiation codons. *Nature*, **343**, 662–665.

BARRETT-LEE, P., TRAVERS, M., LUQMANI, Y. & COOMBES, R.C. (1990). Transcripts for transforming growth factors in human breast cancer: clinical correlates. *Br. J. Cancer*, **61**, 612–617.

BARTEK, J., BÁRIKOVÁ, J., VOJTESEK, B., STASKOVÁ, Z., REJTHAR, A., KOVARIK, J. & LANE, D.P. (1990). Patterns of expression of the p53 tumour suppressor in human breast tissues and tumours *in situ* and *in vitro*. *In. J. Cancer*, **46**, 839–844.

BATES, S.E., DAVIDSON, N.E., VALVERIUS, E.M., FRETER, C.E., DICKSON, R.B., TAM, J.P., KUDLOW, J.E., LIPPMAN, M.E. & SALOMON, D.S. (1988). Expression of transforming growth factor alpha and its messenger ribonucleic acid in human breast cancer: its regulation by estrogen and its possible functional significance. *Mol. Endocrinol.*, **2**, 543–555.

BLOOM, H.J.G. & RICHARDSON, W.W. (1957). Histological grading and prognosis in breast cancer. *Br. J. Cancer*, **11**, 359–377.

CALLAHAN, R. & CAMPBELL, G. (1989). Mutations in human breast cancer: an overview. *J. Natl Cancer Inst.*, **81**, 1780–1786.

CIARDIELLO, F., DONO, R., KIM, N., PERSICO, M.G. & SALOMON, D.S. (1991*a*). Expression of *cripto*, a novel gene of the epidermal growth factor gene family, leads to *in vitro* transformation of a normal mouse mammary epithelial cell line. *Cancer Res.*, **51**, 1051–1054.

CIARDIELLO, F., KIM, N., LISCIA, D.S., BIANCO, C., LIDEREAU, R., MERLO, G., CALLAHAN, R., GREINER, J., SZPAK, C., KIDWELL, W.R., SCHLOM, J. & SALOMON, D.S. (1989). Transforming growth factor α (TGFα) mRNA in human breast carcinomas and TGFα in the effusions of breast cancer patients. *J. Natl Cancer Inst.*, **81**, 1165–1171.

CIARDIELLO, F., KIM, N., MCGEADY, M.L., LISCIA, D.S., SAEKI, T., BIANCO, C. & SALOMON, D.S. (1990*a*). Expression of transforming growth factor alpha (TGFα) in breast cancer. *Ann. Oncol.*, **2**, 169–182.

CIARDIELLO, F., KIM, N., SAEKI, T., DONO, R., PERSICO, M.G., PLOWMAN, G.D., GARRIGUES, J., RADKE, S., TODARO, G.J. & SALOMON, D.S. (1991*b*). Differential expression of epidermal growth factor-related proteins in human colorectal tumors. *Proc. Natl Acad. Sci. USA*, **88**, 7792–7796.

CIARDIELLO, F., MCGEADY, M.L., KIM, N., BASOLO, F., HYNES, N., LANGTON, B.C., YOKOZAKI, H., SAEKI, T., ELLIOTT, J.W., MASUI, H., MENDELSOHN, J., SOULE, H., RUSSO, J. & SALOMON, D.S. (1990*b*). Transforming growth factor-α expression is enhanced in human mammary epithelial cells transformed by an activated c-Ha-*ras* protooncogene but not by the c-*neu* protooncogene, and overexpression of the transforming growth factor-α complementary DNA leads to transformation. *Cell Growth Different.*, **1**, 407–420.

CICCODICOLA, A., DONO, R., OBICI, S., SIMEONE, A., ZOLLO, M. & PERSICO, M.G. (1989). Molecular characterization of a gene of the 'EGF family' expressed in undifferentiated human NTERA2 teratocarcinoma cells. *EMBO J.*, **8**, 1987–1991.

COOK, P.W., MATTOX, P.A., KEEBLE, W.W., PITTELKOW, M.R., PLOWMAN, G.D., SHOYAB, M., ADELMAN, J.P. & SHIPLEY, G.D. (1991). A heparin sulfate-regulated human keratinocyte autocrine factor is similar or identical to amphiregulin. *Mol. Cell. Biol.*, **11**, 2547–2557.

COOK, P.W., PITTELKOW, M.R., KEEBLE, W.W., GRAVES-DEAL, R., COFFEY, Jr, R.J. & SHIPLEY, G.D. (1992). Amphiregulin messenger RNA is elevated in psoriatic epidermis and gastrointestinal carcinomas. *Cancer Res.*, **52**, 3224–3227.

DAVIDSON, N.E. & LIPPMAN, M.E. (1989). The role of estrogens in growth regulation of breast cancer. *Crit. Rev. Oncogen.*, **1**, 89–111.

DONO, R., MONTUORI, N., ROCCHI, M., DE PONTI-ZILLI, L., CICCODICOLA, A. & PERSICO, M.G. (1991). Isolation and characterization of the CRIPTO autosomal gene and its X-linked related sequence. *Am. J. Hum. Genet.*, **49**, 555–565.

DUBLIN, E.A., BARNES, D.M., WANG, D.Y., KING, R.J.B. & LEVIISON, D.A. (1993). TGF alpha and TGF beta expression in mammary carcinoma. *J. Pathol.*, **170**, 15–22.

FINZI, E., HARKINS, R. & HORN, T. (1991). TGF-α is widely expressed in differentiated as well as hyperproliferative skin epithelium. *J. Invest. Dermatol.*, **96**, 328–332.

GASPARINI, G., BEVILACQUA, P., POZZA, F., MELI, S., BORACCHI, P., MARUBINI, E. & SAINSBURY, J.R.C. (1992). Value of epidermal growth factor receptor status compared with growth fraction and other factors for prognosis in early breast cancer. *Br. J. Cancer*, **66**, 970–976.

GULLICK, W.J. (1990). Growth factors and oncogenes in breast cancer. *Prog. Growth Factor Res.*, **2**, 1–13.

HIGASHIYAMA, S., LAU, K., BESNER, G.E., ABRAHAM, J.A. & KLAGSBRUN, M. (1992). Structure of heparin-binding EGF-like growth factor. *J. Biol. Chem.*, **267**, 6205–6212.

HORAK, E., SMITH, K., BROMLEY, L., LEJEUNE, S., GREENALL, M., LANE, D. & HARRIS, A.L. (1991). Mutant p53, EGF receptor and c-*erb*B-2 expression in human breast cancer. *Oncogene*, **6**, 2277–2284.

IMAMURA, T., ENGLEKA, K., ZHAN, X., TOKITA, Y., FOROUGH, R., ROEDER, D., JACKSON, A., MAIER, J.A.M., HIA, T. & MACIAG, T. (1990). Recovery of mitogenic activity of a growth factor mutant with a nuclear translocation sequence. *Science*, **249**, 1567–1570.

JHAPPAN, C., STAHLE, C., HARKINS, R.N., FAUSTO, N., SMITH, G.H. & MERLINO, G.T. (1990). TGFα overexpression in transgenic mice induces liver neoplasia and abnormal development of the mammary gland and pancreas. *Cell*, **61**, 1137–1146.

JOHNSON, G.R., KANNAN, B., SHOYAB, M. & STROMBERG, K. (1993). Amphiregulin induces tyrosine phosphorylation of the epidermal growth factor receptor and $p185^{erbB2}$. *J. Biol. Chem.*, **208**, 2924–2931.

JOHNSON, G.R., SAEKI, T., AUERSPERG, N., GORDON, A.W., SHOYAB, M., SALOMON, D.S. & STROMBERG, K. (1991). Response to and expression of amphiregulin by ovarian carcinoma and normal ovarian surface epithelial cells: nuclear localization of endogenous amphiregulin. *Biochem. Biophys. Res. Commun.*, **180**, 481–488.

JOHNSON, G.R., SAEKI, T., GORDON, A.W., SHOYAB, M., SALOMON, D.S. & STROMBERG, K. (1992). Autocrine action of amphiregulin in a colon carcinoma cell line and immunocytochemical localization of amphiregulin in human colon. *J. Cell Biol.*, **118**, 742–751.

KENNEY, N., JOHNSON, G., SELVAM, P. KIM, N., QI, C.-F., SAEKI, T., BRANDT, R., WALLACE-JONES, B., CIARDIELLO, F., SHOYAB, M., PLOWMAN, G., DAY, A., SALOMON, D.S. & NORMANNO, N. (1993). Transforming growth factor α (TGFα) and amphiregulin (AR) as autocrine growth factors in nontransformed, immortalized 184A1N4 human mammary epithelial cells. *Mol. Cell Different.*, **1**, 163–184.

KIMURA, H. (1993). Schwannoma-derived growth factor must be transported into the nucleus to exert its mitogenic activity. *Proc. Natl Acad. Sci. USA*, **90**, 2165–2169.

KLIJN, J.G.M., BERNS, P.M.J.J., SCHMITZ, P.I.M. & FOEKENS, J.A. (1992). The clinical significance of epidermal growth factor receptor (EGF-R) in human breast cancer: a review on 5232 patients. *Endocrinol. Rev.*, **13**, 3–17.

KUNIYASU, H., YOSHIDA, K., YOKOZAKI, H., YASUI, W., ITO, H., TOGE, T., CIARDIELLO, F., PERSICO, M.G., SAEKI, T., SALOMON, D.S. & TAHARA, E. (1991). Expression of *cripto*, a novel gene of the epidermal growth factor family, in human gastrointestinal carcinomas. *Jpn J. Cancer Res.*, **82**, 969–973.

LEJEUNE, S., LEEK, R., HORAK, E., PLOWMAN, G., GREENALL, M. & HARRIS, A.L. (1993). Amphiregulin, epidermal growth factor receptor, and estrogen receptor expression in human primary breast cancer. *Cancer Res.*, **53**, 3597–3602.

LI, S., PLOWMAN, G.D., BUCKLEY, S.D. & SHIPLEY, G.D. (1992). Heparin inhibition of autonomous growth implicates amphiregulin as an autocrine growth factor for normal human mammary epithelial cells. *J. Cell Physiol.*, **153**, 103–111.

LUNDY, J., CHUSS, A., STANICK, D., MCCORMACK, E.S., KRAMER, S. & SORVILLO, J.M. (1991). Expression of *neu* protein, epidermal growth factor receptor, and transforming growth factor alpha in breast cancer. *Am. J. Pathol.*, **138**, 1527–1534.

MASSAGUE, J. (1990). Transforming growth factor-α: a model for membrane-anchored growth factors. *J. Biol. Chem.*, **265**, 21393–21396.

MATSUI, Y., HALTER, S.A., HOLT, J.T., HOGAN, B.L.M. & COFFEY, R.J. (1990). Development of mammary hyperplasia and neoplasia in MMTV-TGFα transgenic mice. *Cell*, **61**, 1137–1146.

MAZARS, R., SPINARDI, L., BENCHEIKH, M., SIMONY-LAFONTAINE, J., JEANTEUR, P. & THEILLET, C. (1992). p53 mutations occur in aggressive breast cancer. *Cancer Res.*, **52,** 3918–3923.

MERLO, G.R., VENESIO, T., BERNARDI, A., CANALE, L., GAGLIA, P., LAURO, D., CAPPA, A., CALLAHAN, R. & LISCIA, D.S. (1992). Loss of heterozygosity on chromosome 17p13 in breast carcinomas identifies tumors with high proliferation index. *Am. J. Pathol.*, **140,** 215–223.

MIZUKAMI, Y., NONOMURA, A., YAMADA, T., KURUMAYA, H., HAYASHI, M., KOYASAKI, N., TANIYA, T., NOGUCHI, M., NAKAMURA, S. & MATSUBARA, F. (1990). Immunohistochemical demonstration of growth factors, TGF-α, TGF-β, IGF-I and *neu* oncogene product in benign and malignant human breast tissues. *Anticancer Res.*, **10,** 1115–1126.

MODRELL, B., MCDONALD, V.L. & SHOYAB, M. (1992). The interaction of amphiregulin with nuclei and putative nuclear localization sequence binding proteins. *Growth Factors*, **7,** 305–314.

MURPHY, L.C. & DOTZLAW, H. (1989). Regulation of transforming growth factor α and transforming growth factor-β messenger ribonucleic acid abundance in T47D human breast cancer cells. *Mol. Endocrinol.*, **3,** 611–616.

NICHOLSON, S., HALCROW, P., SAINSBURY, J.R.C., AGNUS, J.R., CHAMBEERS, P., FARNDON, J.R. & HARRIS, A.L. (1988). Epidermal growth factor receptor (EGFr) status associated with failure of primary endocrine therapy in elderly postmenopausal patients with breast cancer. *Br. J. Cancer*, **58,** 810–814.

NORMANNO, N., SAEKI, T., BIANCO, C., JOHNSON, G., KENNEY, N., KIM, N., CIARDIELLO, F. & SALOMON, D.S. (1992). Expression of amphiregulin (AR) in oncogene transformed human mammary epithelial cells. *Proc. Am. Assoc. Cancer Res.*, **33,** 271.

NORMANNO, N., QI, C.-F., GULLICK, W.J., PERSICO, G., YARDEN, Y., WEN, D., PLOWMAN, G., KENNEY, N., JOHNSON, G., KIM, N., BRANDT, R., MARTINEZ-LACACI, I., DICKSON, R.B. & SALOMON, D.S. (1993). Expression of amphiregulin, cripto-1, and heregulin α in human breast cancer cells. *Int. J. Oncol.*, **2,** 903–911.

OSBORNE, C.K. & ARTEAGA, C.L. (1990). Autocrine and paracrine growth regulation of breast cancer: clinical implications. *Breast Cancer Res. Treat.*, **15,** 3–11.

OSBORNE, R.J., MERLO, G.R., MITSUDOMI, T., VENESIO, T., LISCIA, D.S., CAPPA, A.P.M., CHIBA, I., TAKAHASHI, T., NAU, M.M., CALLAHAN, R. & MINNA, J.D. (1991). Mutations in the *p53* gene in primary human breast cancers. *Cancer Res.*, **51,** 6194–6198.

PLOWMAN, G.D., GREEN, J.M., MCDONALD, V.L., NEUBAUER, M.G., DISTECHE, C.M., TODARO, G.J. & SHOYAB, M. (1990). The amphiregulin gene encodes a novel epidermal growth factor-related protein with tumor-inhibitory activity. *Mol. Cell. Biol.*, **10,** 1969–1981.

POLLER, D.N., HUTCHINGS, C.E., GALEA, M., BELL, J.A., NICHOLSON, R.A., ELSTON, C.W., BLAMEY, R.W. & ELLIS, I.O. (1992). p53 protein expression in human breast carcinoma: relationship to expression of epidermal growth factor receptor. c-*erb*B-2 protein overexpression, and estrogen receptor. *Br. J. Cancer*, **66,** 583–588.

POWELL, P.P. & KLAGSBRUN, M. (1991). Three forms of rat basic fibroblast growth factor are made from a single mRNA and localize to the nucleus. *J. Cell Physiol.*, **148,** 202–210.

RAKOWICZ-SZULCZYNSKA, E.M. (1993). Identification of the cell surface and nuclear receptors for NGF in a breast carcinoma cell line. *J. Cell Physiol.*, **154,** 64–70.

SAEKI, T., STROMBERG, K., QI, C.-F., GULLICK, W.J., TAHARA, E., NORMANNO, N., CIARDIELLO, F., KENNEY, N., JOHNSON, G.R. & SALOMON, D.S. (1992). Differential immunohistochemical detection of amphiregulin and cripto in human normal colon and colorectal tumors. *Cancer Res.*, **52,** 3467–3473.

SAINSBURY, R. (1990). Epidermal growth factor receptors and prognosis in breast cancer. *Path. Biol.*, **38,** 771–772.

SALOMON, D.S., KIM, N., SAEKI, T. & CIARDIELLO, F. (1990). Transforming growth factor-α: an oncodevelopmental growth factor. *Cancer Cells*, **2,** 389–397.

SALOMON, D.S., DICKSON, R.B., NORMANNO, N., SAEKI, T., KIM, N., KENNEY, N. & CIARDIELLO, F. (1992). Interaction of oncogenes and growth factors in colon and breast cancer. In *Current Perspectives on Molecular and Cellular Oncology*, Vol. 1, part B, Spandidos, D.A. (ed.) pp. 211–260. JAI Press: London.

SANTHANAM, U., RAY, A. & SEHGAL, P.B. (1991). Repression of the interleukin 6 gene promoter by p53 and the retinoblastoma susceptibility gene product. *Proc. Natl Acad. Sci. USA*, **88,** 7605–7609.

SPORN, M.B. & ROBERTS, A.B. (1992). Autocrine secretion – 10 years later. *Ann. Intern. Med.*, **117,** 408–414.

TRAVERS, M.T., BARRETT-LEE, P.J., BERGER, U., LUGMANI, Y.A., GAZET, J.-C., POWELS, T.J. & COOMBES, C.R. (1988). Growth factor expression in normal, benign and malignant breast tissue. *Br. Med. J.*, **296,** 1621–1625.

ULLRICH, S.J., ANDERSON, C.W., MERCER, W.E. & APPELLA, E. (1992). The p53 tumor suppressor protein, a modulator of cell proliferation. *J. Biol. Chem.*, **267,** 15259–15262.

UMEKITA, Y., ENOKIZONO, N., SAGARA, Y., KURIWAKI, K., TAKASAKI, T., YOSHIDA, A. & YOSHIDA, H. (1992). Immunohistochemical studies on oncogene products (EGF-R, c-*erb*B-2) and growth factors (EGF, TGF-α) in human breast cancer: their relationship to oestrogen receptor status, histological grade, mitotic index and nodal status. *Virchows Arch. [A]*, **430,** 345–351.